# Application of the analytic hierarchy approach to the risk assessment of Zika virus disease transmission in Guangdong Province, China

**DOI:** 10.1186/s12879-016-2170-2

**Published:** 2017-01-13

**Authors:** Xing Li, Tao Liu, Lifeng Lin, Tie Song, Xiaolong Du, Hualiang Lin, Jianpeng Xiao, Jianfeng He, Liping Liu, Guanghu Zhu, Weilin Zeng, Lingchuan Guo, Zheng Cao, Wenjun Ma, Yonghui Zhang

**Affiliations:** 1 Guangdong Provincial Institute of Public Health, Guangdong Provincial Center for Disease Control and Prevention, No. 160 Qunxian Road, Panyu District, Guangzhou, 511430 China; 2 Guangdong Provincial Center for Disease Control and Prevention, No. 160 Qunxian Road, Panyu District, Guangzhou, 511430 China; 3 Guangzhou Institute of Geochemistry, Chinese Academy of Sciences, No. 511 Kehua Street, Tianhe District, Guangzhou, 510640 China

**Keywords:** Zika virus, Risk assessment, Transmission, China

## Abstract

**Background:**

An international spread of Zika virus (ZIKV) infection has attracted global attention in 2015. The infection also affected Guangdong province, which is located in southern China. Multiple factors, including frequent communication with South America and Southeast Asia, suitable climate (sub-tropical) for the habitat of *Aedes* species, may increase the risk of ZIKV disease transmission in this region.

**Methods:**

An analytic hierarchy process (AHP) method was used to develop a semi-quantitative ZIKV risk assessment model. After selecting indicators, we invited experts in related professions to identify the index weight and based on that a hierarchical structure was generated. Then a series of pairwise comparisons were used to determine the relative importance of the criteria. Finally, the optimal model was established to estimate the spatial and seasonal transmission risk of ZIKV.

**Results:**

A total of 15 factors that potentially influenced the risk of ZIKV transmission were identified. The factor that received the largest weight was epidemic of ZIKV in Guangdong province (combined weight [CW] =0.37), followed by the mosquito density (CW = 0.18) and the epidemic of DENV in Guangdong province (CW = 0.14). The distribution of 123 districts/counties’ RIs of ZIKV in Guangdong through different seasons were presented, respectively.

**Conclusions:**

Higher risk was observed within Pearl River Delta including Guangzhou, Shenzhen and Jiangmen, and the risk is greater in summer and autumn compared to spring and winter.

## Background

Zika virus (ZIKV) belongs to the virus family *Flaviviridae* and genus *Flavivirus*, which was first identified from a rhesus monkey in the Zika Forest of Uganda in 1947 [1]. The first documented outbreak of ZIKV was in 2007 on Yap Island, Micronesia [2]. Between 2013 and 2014, a large ZIKV epidemic occurred in French Polynesia, followed by rapid spread to countries in Oceania [3]. In 2015, an international spread of ZIKV infection has attracted global attention, as a huge epidemic of ZIKV infection in Brazil was considered to be associated with a remarkable increase of microcephaly cases [4]. A recent scientific consensus support that ZIKV is a cause of microcephaly [5, 6], and consequently the World Health Organization (WHO) issued ZIKV infection and its associated congenital and other neurological disorders as Public Health Emergency of International Concern (PHEIC) [7]. As of August 10, 2016, continuing transmission of ZIKV has been reported in 69 countries and territories [8], mostly in the Americas and some island countries.

Similar to dengue virus (DENV) and chikungunya virus (CHIKV), ZIKV shares self-limiting clinical signs and symptoms [9] and is also transmitted by the common mosquito vectors, namely *Aedes* species. However vaccine of ZIKV is not available yet; therefore it is of utmost importance to identify the transmission risk of ZIKV.

Guangdong province is located in southern China, which is adjacent to the Hong Kong and Macao Special Administrative Region, and has frequent economic and cultural communication with Southeast Asia and South America where ZIKV is endemic [10, 11]. These factors would increase the risk of ZIKV importation. For example, about 300,000 people from Enping county, Guangdong province worked in Venezuela [12]. As July 31, 2016 there were a total of 14 imported ZIKV cases in Guangdong, most of them are migrate workers returned from Venezuela to Enping county [13]. Moreover, Guangdong has a hot and humid sub-tropical climate, which is suitable for the habitat of *Aedes* species, including *Aedes aegypti* and *Aedes albopictus. Aedes*-related virus, e.g., dengue is a serious public health concern in Guangdong Province. Dengue cases have been reported each year for the past 27 years in Guangdong Province, and a recorded historical peak occurred in 2014 [14]. In addition, densely population of the Pearl River Delta in Guangdong province (e.g., Guangzhou, Shenzhen and Foshan) makes it a highly susceptible place for transmission of infectious disease.

The analytic hierarchy process (AHP) was developed Saaty [15] in the 1970s, which incorporates both quantitative and qualitative criteria into analyzing decision problems. It has been widely used to analysis environmental impact assessments [16, 17] and infectious diseases [18–20]. As a semi-quantitative method, AHP offers accurate assessment of risk factors and spatial/seasonal distribution for infectious diseases, which could help to provide precise prevention and control strategies.

To cope the forthcoming potential ZIKV transmission risk at county level, it is fruitful to understand the actual risk of ZIKV epidemic in a semi-quantitative manner. The present study aimed to assess the spatial and seasonal distribution of ZIKA transmission risk by AHP in Guangdong province, China.

## Methods

### Study design

We used AHP to develop a semi-quantitative ZIKV risk assessment model. First, indicators were selected by reference to citations and expert consultations. Then we invited experts to identify the index weight, subsequently generated a hierarchical structure and collected related data. After that, a series of pairwise comparisons were used to determine the relative importance of the criteria relative to ZIKV transmission, which were then combined into a numerical score using a weighting process that accounts for direct and indirect comparisons [15, 21]. The approach allows transparent judgments based on numerical scores. Finally, the optimal model of risk of ZIKV transmission based on this study was developed for future reference.

### Risk assessment framework

We employed a framework in which risk was a function of natural, mosquito-borne, endemic, economic and social elements, and developed indicators of all these dimensions. The final risk index (RI) for each district/county was calculated by summing up natural risk (NR), mosquito-borne risk (MR), endemic risk (ER), economic and social risk (ESR) [18].

$$ {\mathrm{RI}}_{\mathrm{j}}={\mathrm{NR}}_{\mathrm{j}}+{\mathrm{MR}}_{\mathrm{j}}+{\mathrm{ER}}_{\mathrm{j}}+{\mathrm{ESR}}_{\mathrm{j}} $$

Where RI_j_ indicates the overall RI to Zika virus in district/county j. It is estimated mathematically combining natural, mosquito-borne endemic, economic and social elements. NR, MR, ER, ESR in district/county j are expressed as NR_j_, MR_j_, ER_j_, ESR_j_, respectively.

Because natural and mosquito-borne elements vary among different seasons, we calculate RIs in spring (March to May), summer (June to August), autumn (September to November) and winter (December to February), respectively.

### Indicator selection for each dimension

An indicator pool was generated with reference to a range of existing studies [18, 22, 23] and consultations with experts. First, we searched related literature databases, including MEDLINE, PubMed, and China National Knowledge Infrastructure (CNKI). Second, two authors independently selected indicators, and minor discrepancies were resolved by discussion. Meanwhile, expert consultations were also conducted to collect risk indicators. Finally, all collected indicators were gathered to generate a primary indicator pool. Experts from the fields of public health, meteorology, and social sciences were invited to select appropriate indicators for each dimension from the indicator pool based on the following three principles: 1) indicators should sensitively reflect the risk of ZIKV; 2) indicators should be easily implemented in practical work and have no limits imposed by data availability; 3) indicators should reflect being used in existing studies of other countries and regions. After preliminary selection of all indicators, experts discussed the collective suite of indicators, deleted indicators with poor representation or high correlations, and improved indicators that required some modification to make them appropriate for this study.

### Data collection

Indicators were obtained from the National Sixth Census [24], Guangdong Statistical Yearbook [25], and Health Statistics Year book of Guangdong Province [26], Guangdong Provincial Meteorological Bureau, Guangzhou Institute of Geochemistry, Chinese Academy of Sciences (GIG) and Guangdong Provincial Center for Disease Control and Prevention (GDCDC).

### Standardization and weight determination of each indicator

Prior to the index calculation, all individual indicators were standardized to remove potential issues associated with using indicators measured at different scales. Standardization was undertaken with reference to the following formula [23]:

$$ \mathrm{S}\mathrm{t}\mathrm{d}\left({\mathrm{I}}_{\mathrm{i}\mathrm{j}}\right)={\mathrm{I}}_{\mathrm{i}\mathrm{j}}/{ \max}_{\mathrm{i}} $$

In which, Std(I_ij_) is the standardized indicator i for district/county j, I_ij_ is the unstandardized indicator i for district/county j, and max_i_ is the maximum value of indicator i among all districts/counties. Using this standardization approach, each individual indicator was rescaled into a common measurement scale that ranged between 0 and 1.

Before calculating the standardized score of each dimension, AHP was employed to determine the weight of each indicator.

### Experts’ judgment matrix and calculation of consistency ratio (CR)

Fourteen stakeholder experts from epidemiology, infectious disease control, mosquito-borne disease, public health, geographic information, entry-exit inspection and quarantine fields were invited to determine the relative importance of all indicators in each dimension. Then the weight for each indicator was generated based on the relative importance in each dimension of the RI. An expert could subjectively judge the relative importance between indicators following a 1-9 fundamental scale. According to that scale, score of 1 was given to criteria that had equal importance. Scores of 3, 5, 7, and 9 denoted weakly, strongly, very strongly, and absolutely more important, respectively. Scores of 2, 4, 6, and 8 were used when slight differences existed between criteria.

A judgment matrix would be obtained for each dimension from each expert. CR was calculated to evaluate the consistency of the pairwise comparisons. The nearer the CR values were to 0, the greater the consistency of the pairwise comparisons, whereas larger values indicated lower consistencies. The pairwise comparisons were generally considered as consistent if their CR value was <0.10. The final weight for each indicator was an average of the results given by all experts.

### Calculation of RI

In order to apply the weighted indicators to the formula above (section 2.2), separate indicators for RI_j_ were calculated using the following formulas:

$$ {\mathrm{RI}}_{\mathrm{j}}={\mathrm{W}}_{\mathrm{E}1}*{\mathrm{E}}_1+{\mathrm{W}}_{\mathrm{E}2}*{\mathrm{E}}_2+\dots +{\mathrm{W}}_{\mathrm{E}\mathrm{n}}*{\mathrm{E}}_{\mathrm{n}} $$

Where E_1_-E_n_ were indicators for 1 ~ n risk exposures, and W was the weight of each indicator. The higher the RI value, the greater the ZIKV transmission risk is in this district/county. A geographic information system (ArcGIS) was used to display the distribution of RIs among 123 districts/counties of Guangdong Province.

## Results

A total of 15 factors that potentially influenced the risk of ZIKV transmission were identified (Table 1). The fourteen experts gave weight for each indicator individually and then the average weight was calculated (Table 2). The factor that received the largest weight was epidemic of ZIKV in Guangdong province (combined weight [CW] =0.37), followed by the influences of mosquito density (CW = 0.18) and epidemic of DENV in Guangdong province (CW = 0.14). These results indicated that these evaluation factors were considered more important than other factors. The result was consistent with all CRs < 0.10.

**Table 1:** Characteristics of selected indicators in each dimension

Indicators	Sub-indicators	Source	Time of data collection
Mosquito density	Mosquito larvae density	GDCDC	2013-2015
	Mosquito density	GDCDC	2013-2015
Epidemic of Zika in Guangdong province	Imported Zika cases in Guangdong	GDCDC	2016
	Local Zika cases in Guangdong	GDCDC	2016
Epidemic of Dengue Fever in Guangdong province	Previous imported Dengue Fever cases in Guangdong	GDCDC	2013-2015
	Local Dengue Fever cases in Guangdong	GDCDC	2013-2015
Epidemic of Zika in other province of China	Epidemic of Zika in other province of China	GDCDC	2016
Climate and nature factors	Temperature	GMB	2013-2015
	Humidity	GMB	2013-2015
	Rainfall	GMB	2013-2015
	Vegetation density	GIG	2015
Economy and population factors	Population density	National sixth Census	2010
	Economy	Guangdong Statistical Yearbook	2010
Social activity	International activity	Websites	2016
	Zika prevention and control measures	GDCDC	2016

*Abbreviations*: *GDCDC* Guangdong Provincial Center for Disease Control and Prevention, *GIG* Guangzhou Institute of Geochemistry, Chinese Academy of Sciences, *GMB* Guangdong Meteorological Bureau

**Table 2:** Weight of each indicator determined by expert scoring and analytic hierarchy process

Dimension	Indicators	Experts’ weight														Average weight
		A	B	C	D	E	F	G	H	I	J	K	L	M	N	
Mosquito density	Mosquito larvae density	0.32	0.01	0.20	0.06	0.03	0.02	0.01	0.01	0.06	0.02	0.03	0.20	0.12	0.13	0.09
	Mosquito density	0.11	0.06	0.07	0.06	0.14	0.09	0.08	0.06	0.06	0.10	0.05	0.20	0.12	0.13	0.10
Epidemic of Zika in Guangdong province	Imported Zika cases in Guangdong	0.02	0.09	0.04	0.06	0.04	0.08	0.05	0.06	0.25	0.04	0.34	0.03	0.04	0.03	0.08
	Local Zika cases in Guangdong	0.13	0.28	0.12	0.32	0.36	0.39	0.42	0.44	0.25	0.38	0.11	0.15	0.37	0.25	0.28
Epidemic of Dengue Fever in Guangdong province	Previous imported Dengue Fever cases in Guangdong	0.01	0.04	0.06	0.02	0.03	0.01	0.02	0.02	0.01	0.01	0.07	0.02	0.02	0.02	0.03
	Local Dengue Fever cases in Guangdong	0.10	0.20	0.16	0.06	0.20	0.04	0.12	0.12	0.07	0.11	0.09	0.06	0.15	0.15	0.12
Epidemic of Zika in other province of China	Epidemic of Zika in other province of China	0.04	0.02	0.04	0.02	0.02	0.02	0.01	0.03	0.02	0.01	0.03	0.03	0.02	0.05	0.02
Climate and nature factors	Temperature	0.02	0.00	0.02	0.01	0.01	0.00	0.00	0.00	0.00	0.00	0.01	0.00	0.02	0.01	0.01
	Humidity	0.03	0.02	0.07	0.02	0.03	0.02	0.03	0.02	0.02	0.02	0.01	0.01	0.02	0.03	0.03
	Rainfall	0.01	0.00	0.01	0.00	0.00	0.00	0.00	0.01	0.00	0.00	0.00	0.00	0.01	0.02	0.01
	Vegetation density	0.03	0.01	0.09	0.02	0.02	0.05	0.05	0.05	0.06	0.05	0.03	0.02	0.03	0.03	0.04
Economy and population factors	Economy	0.09	0.08	0.05	0.16	0.07	0.23	0.09	0.10	0.13	0.16	0.15	0.21	0.03	0.09	0.12
	Population density	0.03	0.02	0.02	0.02	0.00	0.01	0.01	0.01	0.01	0.01	0.01	0.02	0.01	0.01	0.01
Social activity	International activity	0.01	0.03	0.01	0.13	0.00	0.03	0.01	0.02	0.02	0.01	0.01	0.01	0.00	0.04	0.02
	Zika prevention and control measures	0.05	0.15	0.05	0.03	0.03	0.01	0.09	0.06	0.05	0.06	0.06	0.04	0.03	0.01	0.05
CR		0.08	0.08	0.06	0.07	0.09	0.09	0.05	0.07	0.07	0.05	0.04	0.09	0.09	0.09	

*Abbreviations*: *CR* consistency ratio

**Fig. 1:**
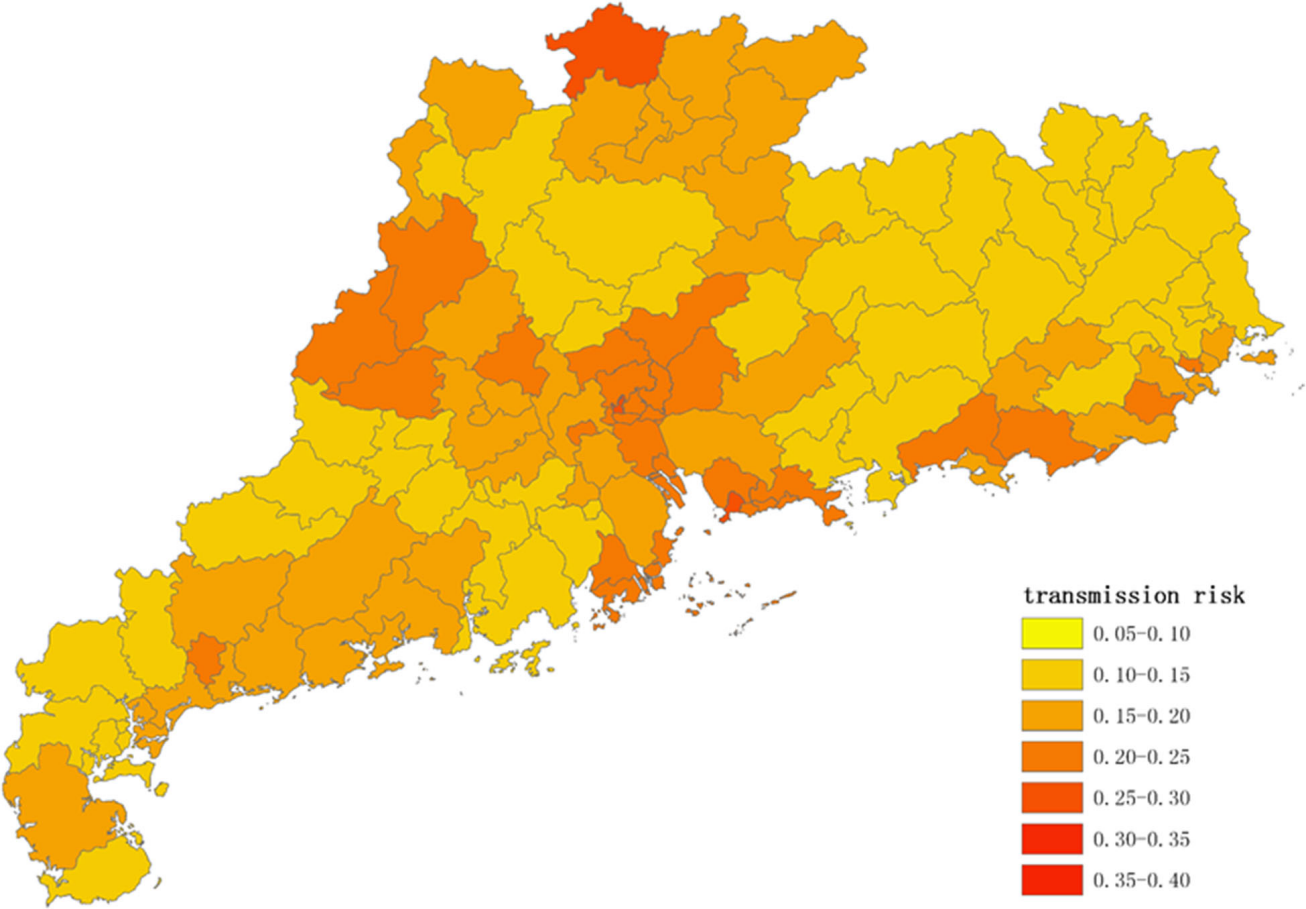
The distribution of risk to ZIKV transmission among 123 counties/districts in Guangdong Province (analytic hierarchy process method) in spring (March to May)

**Fig. 2:**
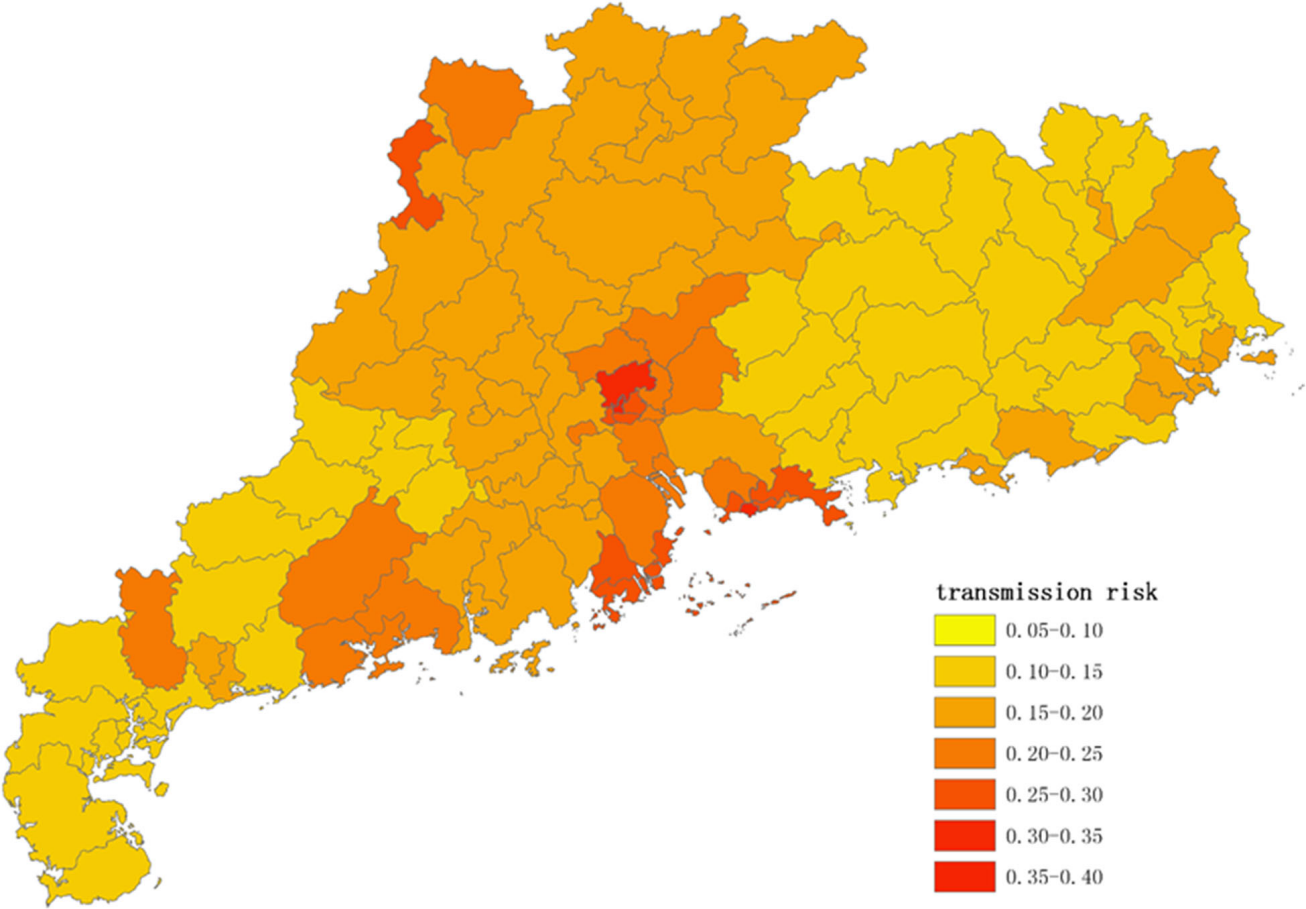
The distribution of risk to ZIKV transmission among 123 counties/districts in Guangdong Province (analytic hierarchy process method) in summer (June to August)

**Fig. 3:**
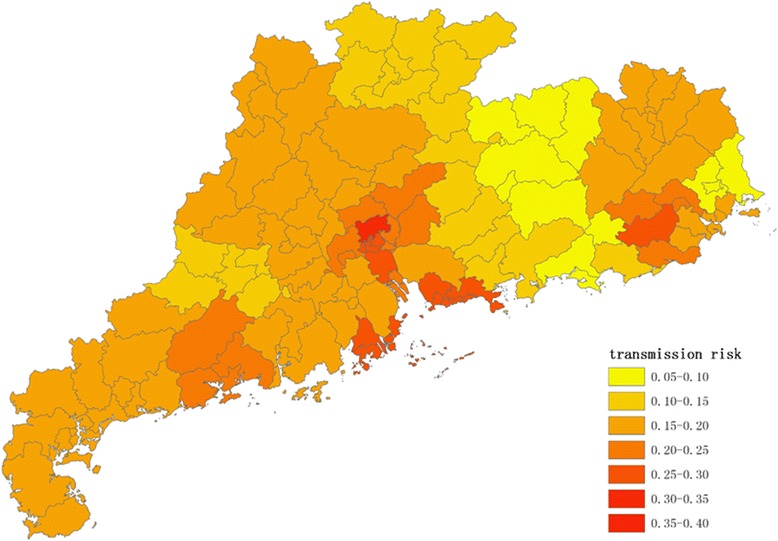
The distribution of risk to ZIKV transmission among 123 counties/districts in Guangdong Province (analytic hierarchy process method) in autumn (September to November)

**Fig. 4:**
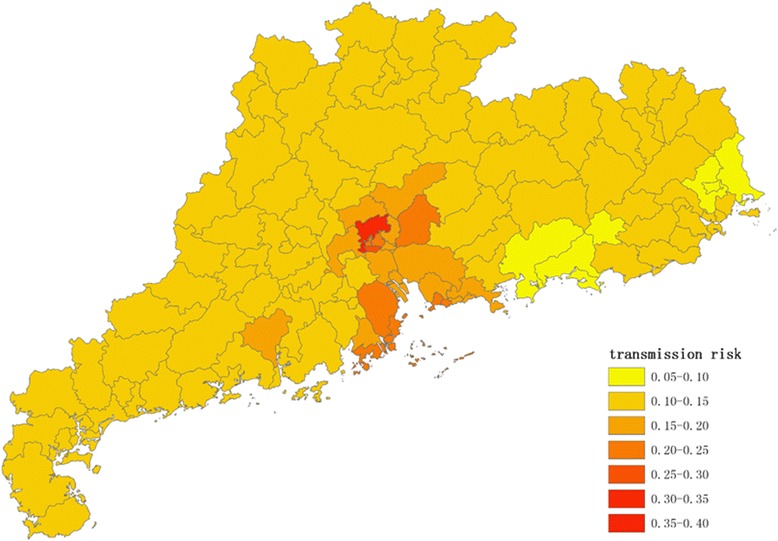
The distribution of risk to ZIKV transmission among 123 counties/districts in Guangdong Province (analytic hierarchy process method) in winter (December to February)

Figures 1, 2, 3 and 4 show the distribution of 123 districts/counties’ RIs of ZIKV in Guangdong Province during spring (March to May), summer (June to August), autumn (September to November) and winter (December to February), respectively. The overall RI in spring is relatively low, ranging from 0.10 to 0.26. During summer, the average score of RI was 0.18 with the highest in Yuexiu district of Guangzhou (RI = 0.38) and the lowest in Zijin county of Heyuan city (RI = 0.08). The RIs were higher in Pearl River Delta including Shenzhen, Guangzhou, Zhuhai and Enping county of Jiangmen city (Fig. 2). In autumn (Fig. 3), top ten counties/districts with highest ZIKV RI all locate in Guangzhou and Shenzhen. In winter (Fig. 4), the overall risk of ZIKV will be decreasing. The lowest RI was 0.07 in the urban area of Shanwei, while a highest risk was in Baiyun district, Guangzhou (RI = 0.35).

## Discussion

AHP is one of the most commonly used utility-based methods for multi-attribute decision-making. It uses objective mathematics to process the subjective and personal preferences in decision making [27]. AHP has been used in risk assessment of emerging infectious diseases (EIDs) [18] including Dengue [20]. Tu et al. [18] assessed the likelihood of epidemic of EIDs by generating a 3-layer hierarchy with 14 evaluation factors. He et al. [20] used AHP to assess the risk of local transmission of Dengue caused by introduced cases.

Using an AHP method, the present study mainly focused on the analysis of the seasonal county-specific risk of ZIKV transmission in Guangdong province, China. We evaluated the contribution of related factors to the transmission and estimated the transmission risk by time and space.

For imported risk, even though the number of countries and regions affected by ZIKV is still rising, the endemic was mainly concentrated in the America, and the increasing speed has slowed down [8]. But some Pacific island countries are still with high incidence [8]. Because of the tropical location, ZIKV epidemic could continue all the year in Pacific island countries, so the pressure of imported cases from these areas will last for a long time. In Guangdong, fourteen imported cases have been reported in Guangdong province update to July 31, 2016. All of these cases come back from the America to Enping county, Jiangmen city. Therefore local authorities put great efforts on following up those who come back from the America, and 1069 returnees were tracked from February 1 to May 15, 2016. The other potential source of imported Zika cases is Southeast Asia. Indeed, several countries in Southeast Asia have reported sporadic cases in 2016, and no local transmission was found yet [28, 29]. In the view of this, it might not be high risk of imported cases of Zika cases from Southeast Asia.

The risk in Pearl River Delta is higher than the other part of Guangdong. Throughout Guangdong province the counties/districts of Shenzhen, Guangzhou, Zhuhai and Enping have the highest risk of ZIKV transmission. From the aspect of season, it is necessary to implement surveillance to detect human cases of local mosquito-borne transmission of ZIKV during peak mosquito-biting season (summer and autumn). Enhancement of mosquito control is also essential to detect local transmission.

For local transmission, areas with imported cases of ZIKV disease and local circulation of *Aedes* mosquitoes are at increased risk for ZIKV transmission. Several main influencing factors contribute to local ZIKV transmission risk. First, conditions of mosquitoes including larvae and adult mosquito rate could affect the transmission of ZIKV. Experts participated in AHP confirmed that mosquito density is a key factor for ZIKV transmission (CW = 0.18). Based on infectious and transmission experiment, *Aedes aegypti*, *Ae. albopictus* may be potential vectors of ZIKV in Mainland China (data not published yet). Therefore counties/districts with high *Aedes* mosquito density are in high ZIKV transmission risk. Secondly, population density, urbanization rate may be alternative. For cities like Guangzhou and Shenzhen, the concentrated population and frequently human exchange could raise the transmission possibility. Thirdly, prevention and control measures to improve the public consciousness in local areas may prevent the transmission of ZIKV. Health and Family Planning Commission of Guangdong issued provincial surveillance protocol of vectors on February 9, 2016 and ZIKV prevention and control guideline on March 11, 2016. In April 28 health campaign month was implemented in Guangdong Province. In addition, international activities (e.g., Brazil Olympic Games and the China Export Commodities Fair) may have an influence on ZIKV in Guangdong province. But according to WHO, there is a very low risk of further international spread of ZIKV as a result of the Olympic and Paralympic Games as Brazil would be hosting the Games during the Brazilian winter (August 5 to September 15) when the intensity of autochthonous transmission of ZIKV will be minimal [30].

For ZIKV, Nah et al. [22] employed a mathematical survival analysis model to analyze the risk for 198 countries, while Tu et al. [18] conducted a qualitative and quantitative risk assessment to evaluate the risk of importation and autochthonous transmission in Mainland China. However, since there is no local transmitted ZIKV case in Guangdong province and we have no access to airline transportation network data between counties of Guangdong province and ZIKV epidemic countries, it’s not appropriate to use mathematical survival analysis model. Second, to improve Tu et al.’s method [18], we chose AHP to make objective evaluation of the risk factor, which could be used to solicit input from stakeholders. To avoid subjective or unreasonable weight assignment, the weights of indicators were determined by expert scoring and CR was calculated to evaluate the consistency of the pairwise comparisons in our study. Therefore this study could provide a reference for the prevention and control of ZIKV in Guangdong province and could potentially benefit ZIKV monitoring and prediction. It might also increase the public awareness to the risk of ZIKV transmission.

However, limitations should be mentioned. Firstly, there may be substantial disparity in understanding level of AHP among experts; thus it may be difficult to reach an agreement through the modeling. Therefore, we included a detailed introduction and guideline of AHP to all the experts. And if the expert’s judgment matrix can’t pass the consistency test, he would be asked to recheck the logicality of his answers. Secondly, it is possible that criteria with large number of sub-criteria receive more weights, which needs to be considered. To solve this problem, we calculated the CR. All the pairwise comparisons were generally considered as consistent with CR values <0.10. Thirdly, the uncertainty is a potential problem with all multi-criteria models, including AHP. It is hard to evaluate the communication between Guangdong and endemic areas, since the data of entry-exit population from endemic countries and the airline transportation network is not available. Several methods were used to decrease the uncertainty. We excluded those unavailable indicators in the process of indicator selection; and experts from multiple related disciplines (including epidemiology, infectious disease control, mosquito-borne disease, public health, geographic information, entry-exit inspection and quarantine fields) were invited to avoid cognitive bias. In addition, there are very few data and literatures available in the study region for using computational methods to evaluate the uncertainties of the model. The lack of knowledge has made it difficult to perform a timely quantitative assessment on this novel virus. Therefore, region-wide monitoring network of the environment and human health risk should be established. More accurate research could be conducted with the accumulation of data.

## Conclusions

The risk of ZIKV disease transmission was estimated in Guangdong, China. Higher risk was observed within Pearl River Delta including Guangzhou, Shenzhen and Jiangmen. For seasonal distribution, the transmission risk is greater in summer and autumn compared to spring and winter. The local government should preferentially develop strategies to prevent the transmission of ZIKV disease.

## Abbreviations

AHP: Analytic hierarchy process
CHIKV: Chikungunya virus
CNKI: China National Knowledge Infrastructure
CR: Consistency ratio
CW: Combined weight
DENV: Dengue virus
ER: Endemic risk
ESR: Economic risk and social risk
GDCDC: Guangdong Provincial Center for Disease Control and Prevention
GIG: Guangzhou Institute of Geochemistry, Chinese Academy of Sciences
MR: Mosquito-borne risk
NR: Natural risk
PHEIC: Public Health Emergency of International Concern
RI: Risk index
WHO: World Health Organization
ZIKV: Zika virus
